# Targeted Therapy in Locally Advanced and Recurrent/Metastatic Head and Neck Squamous Cell Carcinoma (LA-R/M HNSCC)

**DOI:** 10.3390/cancers8030027

**Published:** 2016-02-26

**Authors:** María José Echarri, Ana Lopez-Martin, Ricardo Hitt

**Affiliations:** Department of Medical Oncology, Hospital Universitario Severo Ochoa, Avenida Orellana s/n, Leganés, 28911 Madrid, Spain; alopezmartin@gmail.com

**Keywords:** SCCHN, signal transduction, EGFR, cetuximab, HNSCC

## Abstract

Surgery and radiotherapy are the standard treatment options for patients with squamous cell carcinoma of the head and neck (SCCHN). Chemoradiotherapy is an alternative for patients with locally advanced disease. In recurrent/metastatic disease and after progression to platin-based regimens, no standard treatments other than best supportive care are currently available. Most SCCHN tumours overexpress the epidermal growth factor receptor (EGFR). This receptor is a tyrosine-kinase membrane receptor that has been implicated in angiogenesis, tumour progression and resistance to different cancer treatments. In this review, we analysed the different drugs and pathways under development to treat SCCHN, especially recurrent/metastatic disease. Until now, the EGFR signalling pathway has been considered the most important target with respect to new drugs; however, new drugs, such as immunotherapies, are currently under study. As new treatments for SCCHN are developed, the influence of therapies with respect to overall survival, progression free survival and quality of life in patients with this disease is changing.

## 1. Introduction

SCCHN is a heterogeneous disease, both anatomically and biologically [1]. This type of tumour can arise in the oral cavity, oropharynx, hypopharynx or larynx, and it is widely accepted that tobacco and alcohol consumption are risks factors for the development of this disease. Over the past few years, infections with the human papillomavirus (HPV) has been identified as another risk factor, especially for oropharynx cancers. This finding has led to the division of SCCHN in two subsets with different clinical and molecular profiles: HPV-positive tumours (generally with a better prognosis and containing non-smoker patients) and HPV-negative tumours (a worse prognosis). So far, in a locally advanced setting, treatments such as surgery, radiation and chemotherapy based on platinum salts are the elective treatments that are used [2]. However, despite these approaches, most of these patients relapse except HPV+ population. Once the disease has relapsed or become metastatic, the median survival is approximately 6–9 months. In this era of personalized medicine, it is necessary to better describe the molecular biology of HNSCC to develop new strategies for treatment and to detect biomarkers that indicate responsiveness to new therapeutic drugs. Although some molecular advances have been made (e.g., whole genome sequencing of HNSCC) [3,4], translation of these advances into clinical practice has been poor. Indeed, the only targeted therapy currently approved for recurrent or metastatic (R/M) HNSCC is the anti-EGF receptor (EGFR) antibody, cetuximab.

## 2. The HNSCC Genome and Molecular Pathways

The cancer Genome Atlas Research Program (TCGA) presented in ASCO 2013 revealed the first whole-exome sequencing of HNSCC. They analysed samples from 279 head and neck cancer patients who were mostly men, 62% of which had cancers located in the oral cavity. Only 36 of the patients (13%) were HPV-positive. The study showed genetic alterations in oncogenes (CCND1, MYC, and HRAS), tumour suppressor genes (NF1, TP53, and CDKN2A), oncogenic pathways (phosphotidylinositol-3 kinase, PI3K) and Tyrosine Kinase Receptors (EGFR, FGFR1, 2, and 3, MET, IGFR, EPHA2, and DDR2) [5]. Mutation, overexpression of the protein or alteration of the expression of the protein resulting from changes the methylation status were the main mechanisms found. These mutation patterns differed by HPV status. HPV-negative tumours were TP53-positive (84%), while HPV-positive tumours were TP53-negative (only 3% were positive), displayed very few alterations in EGFR, and were PI3KCA-positive (56%). PI3KCA was also positive in 34% of HPV-negative tumours, indicating that it is, like TP53, a commonly mutated pathway. In addition, NOTCH1 was mutated in more than 15% of the tumours.

**Figure 1:**
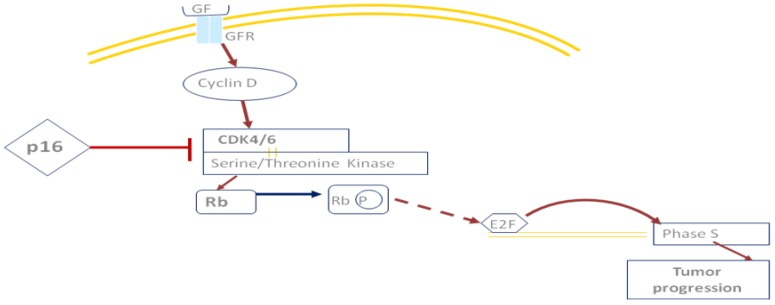
The function of p16/CDKN2A. Cyclin D activates CDK4/6, which phosphorylates Rb, inactivating it. Release of the transcription factor E2F activates cell progression. p16 inhibits CDK4/6 to block cell progression. **CDK:** cyclin-dependent kinase, **Rb**: retinoblastoma.

**Figure 2:**
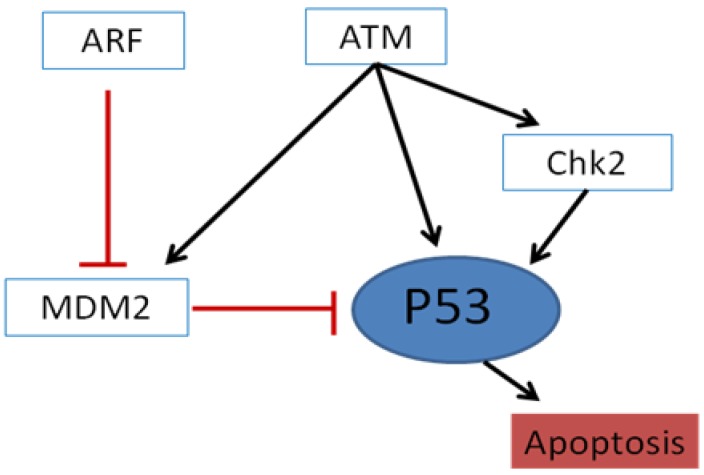
p53 regulation. p53 is a tumour suppressor gene that is activated by a mitogen (via ARF/p14) or genotoxic (ATM) damage. MDM2 inhibits p53 and promotes p53 degradation by ubiquitination.

**Figure 3:**
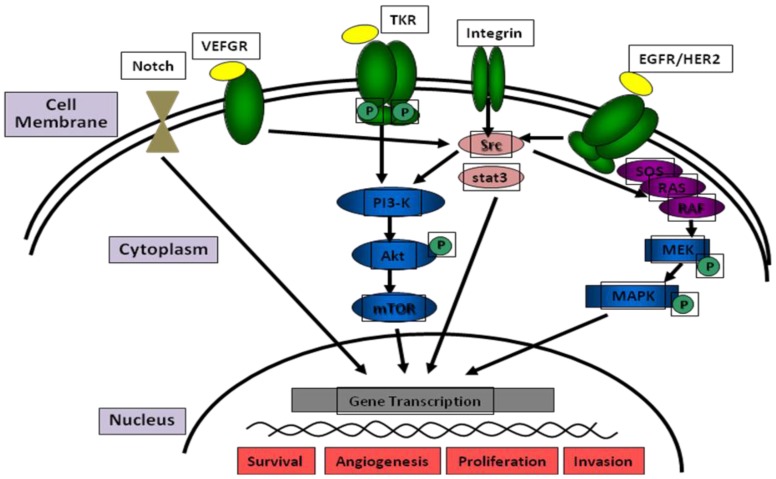
The main pathways involved in HNSCC. TKR: tyrosine kinase receptor, VEFGR: vascular endothelial growth factor receptor, EGFR: epidermal growth factor receptor, HER2: human epidermal growth factor receptor 2, PI3K: phosphatidylinositol 3 kinase, mTOR: mammalian target of rapamycin, MAPK: mitogen-activated protein kinase.

These genomic alterations involved lot of different pathways, including the the CDKN2A/p16 and Rb pathways (Figure 1), p53 and MDM2 pathways (Figure 2), the EGFR pathway and downstream pathways (e.g., RAS, PI3K, and SCR) or the Notch pathway, that affect the cell cycle, disease progression, proliferation, migration and apoptosis [6,7] (Figure 3).

Among all these alterations, four high-frequency alterations are theoretically targetable: EGFR, FGFR, CDKN2A and PIK3CA [8,9].

## 3. Clinical Evidence for Targeted Treatments for HNSCC

### 3.1. EGFR

EGFR remains the only non-chemotherapy molecular target that has been successfully translated into a biological therapy with clinical efficacy [10]. It is supported by high EGFR protein expression (in approximately 90% of HNSCCs). TGF-α and amphiregulin activate EGFR (a member of the ErbB/HER family of tyrosine kinases receptors) to phosphorylate and activate critical proteins, such as those in the PI3K, RAS or Scr kinase pathways, that also control proliferation, angiogenesis and metastasis and the differentiation of epidermal and mesenchymal cells.

The mechanisms employed in therapeutic targets against EGFR include monoclonal antibodies (against the extracellular domain of EGFR), such as cetuximab, panitumumab, zalutumumab and nimotuzumab, and small molecules, including tyrosine kinase inhibitors (TKI) that bind to the intracellular region of EGFR, such as gefitinib, erlotinib, lapatinib, afatinib and dacomitinib. These EGFR inhibitors have been tested in several clinical trials, including phase III trials, with discouraging results, except for cetuximab.

#### 3.1.1. Anti-EGFR Monoclonal Antibodies: Cetuximab

Cetuximab is a monoclonal antibody that binds to EGFR and alters the TK-mediated signal transduction pathway. The drug is active in colon cancer and SCCHN patients. For locally advanced disease, the use of a combination of cetuximab and radiotherapy has shown to benefit survival compared to the use of radiotherapy alone as radical treatment. Cetuximab is an active treatment in platin-refractory patients with recurrent/metastatic disease.

The overexpression of EGFR in SCCHN and histologically normal tissue adjacent to tumour tissues implicates EGFR in SCCHN carcinogenesis. Some evidence suggests that the amplification of the EGFR gene may contribute to EGFR overexpression in malignant tissue [11]. The most common mutated form of EGFR contains a six-exon deletion that encode a 268-amino acid section of its extracellular domain. This mutant, EGFRvIII, is expressed only in cancer cells [11]. EGFRvIII expression has been described in cancers of the brain, lung, breast and prostate [12]. Because mutant EGFR is expressed in cancer cells but not in normal epithelial cells, a mutant EGFR-targeting agent would not interfere with normal EGFR signalling and would therefore have great potential for use as a highly specific targeted approach to treat these cancers. In SCCHN, EGFR is overexpressed in 80% to 90% of tumours, and the clinical relevance of EGFR overexpression as an independent prognostic factor in SCCHN has been well documented [13]. High tumour levels of EGFR are correlated with advanced stage, increased tumour size, decreased survival and decreased sensitivity to radiation treatment [14,15,16,17]. The aberrant functionality of the EGFR network observed in SCCHN provides compelling evidence for a relationship between EGFR and the development and progression of SCCHN and suggests a role for EGFR as a target for cancer therapies.

Cetuximab (IMC-225; Im-Clone Systems, Bridgewater, NJ, USA) [18,19] is a chimeric mouse-human monoclonal antibody that binds EGFR at its extracellular domain and blocks EGF-induced autophosphorylation in EGFR cell lines *in vitro* [20]. It also induces the dimerization and downregulation of EGFR, perturbs cell cycle progression by inducing G1 arrest through an increase in the protein level of p27, an inhibitor of cyclin-dependent kinases, and inhibits tumour-induced angiogenesis [21]. Cetuximab has been shown to have preclinical activity *in vitro* and *in vivo* as both a single agent and in combination with cytotoxic agents and radiotherapy in a wide range of human cancer cell lines, including colorectal, pancreatic, prostate, head and neck and ovarian cancer cells.

In phase I studies [22], doses from 5 to 400 mg/m^2^ have been explored without reaching a maximum tolerated dose (MTD). Pharmacokinetics analyses have shown non-linear behaviour for this drug, with saturation of drug clearance at doses over 200 mg/m^2^. Therefore, the dose regimen selected for phase II-III trials was a loading dose of 400 mg/m^2^ followed by a weekly maintenance dose of 250 mg/m^2^ [22]. Phase I trials revealed favourable tolerability, with the most significant reported toxic effects being an acneiform rash and folliculitis involving the face and upper chest, which occurred in 80% of the patients [23]. Hypersensitivity reactions, although uncommon (<5%), have been reported, with some of them occurring within minutes of the infusion. These were rarely life-threatening. Other adverse effects include asthenia, fever, hypomagnesia and changes in the results of liver function tests.

#### 3.1.2. Cetuximab and Radiotherapy

*In vivo* experiments have demonstrated enhanced antitumour activity in mice treated with cetuximab and radiotherapy [24,25]. In a study by Milas *et al.*, cetuximab improved the efficacy of local tumour irradiation, particularly when multiple injections of cetuximab were administered [25]. Tumour radio-responsiveness was enhanced by a factor of 1.59 by a single dose and by a factor of 3.62 by three doses of cetuximab. Histological analyses of tumours revealed that cetuximab caused a striking increase in central tumour necrosis that was associated with haemorrhage and vascular thrombosis when combined with radiotherapy. In this report, the authors demonstrated that cetuximab greatly enhanced *in vivo* tumour responsiveness to radiation, and this effect was greater than the sum of growth delays caused by the individual treatments.

In another phase I study conducted by Robert *et al.*, a total of sixteen patients were treated using five successive treatment schedules consisting of cetuximab and radiotherapy [22]. Cetuximab was delivered as a loading dose at 100 to 500 mg/m^2^ and was followed by weekly infusions of 100 to 250 mg/m^2^ for 7 to 8 weeks with conventional radiotherapy (RT) or hyperfractionated RT. In this trial, the recommended phase II/III dose was defined as a loading dose of 400 to 500 mg/m^2^ and a maintenance weekly dose of 250 mg/m^2^. The most commonly adverse events were fever, asthenia and skin toxicity (grade 1 to 2 in most patients). Of the 16 included patients, 13 achieved a complete response. This was the first report wherein the activity and safety of cetuximab and radiotherapy were documented. However, in trials wherein cetuximab has been combined with RT and chemotherapy (CT), the results have been contradictory [26]. Thus, in a study by Pfister *et al.* that included patients with SCCHN stage III/IV who were treated with a first line treatment of RT, cisplatin at 100 mg/m^2^ for weeks 1 and 4 and cetuximab (400 mg/m^2^ in week 1 followed by 250 mg/m^2^ during weeks 2 to 10) was closed as a result of significant adverse events, including three deaths (one from myocardial infarction, one from bacteremia and one from atrial fibrillation). There were not significant difference in survival. For this reason, the authors did not recommended this regimen except in a clinical trial setting. In our opinion, a weekly schedule of CT and cetuximab should be explored in combination with radiotherapy to allow this form of CT administration to better manage toxicity compared to the administration of a high dose of cisplatin every three weeks.

The efficacy of cetuximab and radiotherapy was also analysed by Bonner *et al.* [27]. In a multinational randomized phase III trial, radiotherapy alone was compared to RT plus cetuximab in the treatment of locoregionally advanced SCCHN. Patients with stage III/IV head and neck cancer of the oropharynx, hypopharynx or larynx were randomly assigned to treatment with high dose radiotherapy alone (*n* = 213) or high dose RT plus weekly cetuximab (*n* = 211). In this trial, the investigators were required to select one of three radiotherapy-fractionation regimens, including once daily, twice daily or a concomitant boost up to 70–72 Gy. In the group assigned to receive radiotherapy plus cetuximab, administration of intravenous cetuximab was initiated one week before radiotherapy at a loading dose of 400 mg/m^2^ followed by weekly 60-min infusions of 250 mg/m^2^ for the duration of radiotherapy. Patients were stratified according to Karnofsky Performance Status (60 to 80 *vs.* 90 to 100), nodal involvement (N0 *vs.* N+), tumour stage (T1–T3 *vs.* T4) and radiation-fractionation regimen (concomitant boost *vs.* once daily *vs.* twice daily). The primary end point of this study was the duration of control of the locoregional disease. With a median follow-up of 54 months, the median duration of locoregional control was 24.4 months among patients treated with cetuximab and 14.9 months among those that received radiotherapy alone (*p* = 0.005). The median duration of overall survival was 49 months among patients treated with combined therapy and 29.3 months in those treated with RT alone (*p* = 0.03). There was a 26% reduction in the risk of death in the group that received radiotherapy plus cetuximab compared to the group that received radiotherapy alone (hazard ratio, 0.74; 95% confidence interval, 0.57 to 0.97). We emphasize that locoregional control and overall survival were superior in the patients in whom an oropharynx primary tumour was treated with cetuximab/radiotherapy than other in those with other localizations, probably due to HPV prevalence, or in patients who received a concomitant boost of radiotherapy and cetuximab. However, the different arms were well balanced with respect to the primary site of the tumour and the type of radiotherapy, with a concomitant boost of radiotherapy and cetuximab being the best combination schedule. The toxicity profile was similar in both arms, with the exception of an acneiform rash and infusion reactions. Cetuximab did not exacerbate the common toxic effects associated with radiotherapy of the head and neck, including mucositis, xerostomia, dysphagia, pain and performance status deterioration. The results of this phase III trial demonstrated that the combination of cetuximab and RT is better than radiotherapy alone in locally advanced head and neck cancer and that it has a manageable toxicity profile. Therefore, this combination could be the standard treatment in certain settings, such as older patients or those with poor performance status or stage III/IV disease, in whom CT administration cannot be associated with radiotherapy. By other hand, probably there are a set of patients where RT alone is likely best.

Currently, there are several ongoing clinical trials that aim to compare cetuximab/RT with chemoradiotherapy to define the optimal treatment in this set of patients. These trials always take into account the excellent toxicity profile of cetuximab and radiotherapy. Until now, none of these trials has demonstrated a benefit with respect to a combination between cetuximab/chemoradiotherapy, inclusive of those in which there were increased side effects without a benefit in response rate or survival.

#### 3.1.3. Cetuximab as a Single Agent and in Combination with Chemotherapy in Recurrent/Metastatic SCCHN

Every year, 72,000 patients in Europe are diagnosed with recurrent/metastatic SCCHN, for which treatment options are limited. Palliative chemotherapy with cisplatin regimens is the principal option, and it has a median survival time of 6–8 months [28]. No standard second-line treatment after progression while on cisplatin chemotherapy is currently available. The results of a retrospective study by León, Hitt, *et al.* that analysed clinical records from 151 patients with SCCHN refractory to cisplatin chemotherapy showed that most of them had received only best supportive care and that they showed a median survival of 56 days. In this analysis, 28% of the patients received second-line chemotherapy without an objective response, and these had similar survival [29].

An analysis of the results of this retrospective study in combination with the problems that are frequently seen in clinical practice in this set of patients indicates that additional treatment options are urgently required for the management of this disease.

Baselga *et al.* reported the results of a phase II trial in which patients with recurrent/metastatic SCCHN refractory to cisplatin received cetuximab as second-line treatment. In this study, the overall response and disease control rates were 10% and 53%, respectively, and the duration of response was 153 days. Subgroup analysis revealed that there was a trend toward favouring a high response and disease control in older patients, those with higher KP, and those who did not present metastases. In regard to surrogate markers for responsiveness, although significant differences were not observed, there was a trend for patients developing grade 1 or 2 skin reactions in response to treatment (60%) to achieve slightly prolonged times for progression and overall survival compared to patients without skin reactions (39%) [30]. It should be emphasized that there was a low frequency of grade 3/4 adverse events (<15%) in this trial, with skin reactions and acne-like rashes being those most frequently observed. The most interesting aspect of this study was that cetuximab may have reversed resistance to treatment with cisplatin with an acceptable safety profile in this population of patients with poor prognosis for whom there are no current recommended standard treatments.

Herbst *et al.* reported the results of another phase II clinical trial that studied the efficacy and safety of cetuximab administered with cisplatin to patients with refractory metastatic or recurrent SCCHN disease [31]. Following treatment with cisplatin/paclitaxel or cisplatin/fluoruracil, patients with stable or progressive disease received combination therapy with cetuximab and cisplatin. In this trial, up to 20% of the patients with progressive disease (PD) and up to 18% of the patients with stable disease (SD) responded to the combination treatment. The median overall survival was 6.1 and 11.7 months (for PD and SD, respectively). The most common toxicities were anaemia, acne-like skin rash, fatigue and nausea-vomiting. Of these patients, 5% developed a grade 3 or 4 hypersensitivity reaction to cetuximab. The results of this trial confirmed the activity of cetuximab in patients with refractory disease and that prolonged survival was observed in the patients who showed no response to previous therapy with cisplatin.

A phase III, randomised, placebo-controlled clinical trial was conducted by the Eastern Cooperative Oncology Group to determine whether the addition of cetuximab to cisplatin therapy improves progression-free survival in metastatic/recurrent SCCHN compared to cisplatin alone [32]. A total of 121 patients were included in the study, and an objective response was seen in 9.8% of the cisplatin/placebo patients compared to 26.3% of the cisplatin/cetuximab patients (*p* = 0.03). The median progression-free survival time for the control arm was 2.7 months, whereas it was 4.2 months for the cisplatin/cetuximab group (*p* = 0.09). The median overall survival was 8.0 months for patients receiving cisplatin/placebo compared to 9.2 months for patients receiving cisplatin/cetuximab treatment (*p* = 0.21). A significant survival advantage was seen for the development of rash (the hazard ratio for survival by skin toxicity in cetuximab-treated patients was 0.42). In this study, there was a significant benefit in response rate from the addition of cetuximab, albeit progression-free and overall survival were not significantly improved in these patients. The limitations of the study include the limited sample size, the significant drop-off rate in both arms and the fact that the median progression-free survival in the control arm was better than had been projected based on historical experience. All these factors could explain the failure of the primary end-point.

#### 3.1.4. Actual Regimens

An Extreme study to compare cisplatin/fluouracil alone *vs.* the same schedule plus cetuximab was conducted in Europe. In this trial, patients with recurrent/metastatic disease were included in a front line treatment group, and the primary end-point of the study was overall survival. In this trial, 442 patients were included. The addition of cetuximab to platinum-fluouracil significantly prolonged median survival times from 7.4 months in the chemotherapy alone group to 10.1 months in the group that received chemotherapy plus cetuximab (hazard ratio for death, 0.80; 95% confidence interval, 0.64–0.99; *p* = 0.04). The addition of cetuximab also prolonged the median progression-free survival time from 3.3 to 5.6 months (hazard ratio for progression, 0.54; *p* < 0.001) and increased the response rate from 20% to 36% (*p* < 0.001). Moreover, a protocol-defined subgroup analysis showed that the beneficial effects that resulted from adding cetuximab to chemotherapy in overall survival and progression-free survival were evident in nearly all subgroups. This was the first time in over 30 years that the superiority of a new regimen over the standard platinum-based combination chemotherapy was observed. A combination cetuximab and platinum-based chemotherapy is now considered a new standard for the treatment of recurrent/metastatic HNC (reference EXTREME) [33].

Hitt *et al.* reported a phase II trial [34] to test a combination of paclitaxel/cetuximab. Patients received paclitaxel (80 mg/m^2^) and cetuximab (400/250 mg/m^2^) weekly until the disease progressed or unacceptable toxicity was observed. The primary endpoint was the response rate. Among the 46 patients enrolled, the overall response rate was 54% (95% CI 39–69), with 10 (22%) showing complete responses and a disease control rate of 80%. The median progression-free and overall survival times were 4.2 months (95% CI 2.9–5.5 months) and 8.1 months (95% CI 6.6–9.6 months), respectively. Common grade 3/4 adverse events included acne-like rash (24%), asthenia (17%) and neutropenia (13%). Prior chemotherapy and the development of acne-like rash were associated with tumour responsiveness but not survival. No association between tumour EGFR expression or *EGFR* gene copy number and response or survival was found.

The combination of cetuximab and weekly paclitaxel was active and well tolerated by these poor prognosis patients and may therefore be an option for the treatment of medically unfit patients, particularly those for whom platinum is contraindicated, however this is not a standard treatment.

#### 3.1.5. Anti-EGFR non-Cetuximab Monoclonal Antibodies

After good results were observed for cetuximab, the development of new anti-EGFR antibodies was pursued in HNSCC [35,36,37,38]:

*Panitumumab* is a fully human anti-EGFR monoclonal antibody that was approved in combination with chemotherapy for the treatment of metastatic RAS wild-type colorectal cancer. In R/M HNSCC, the phase III SPECTRUM trial [39] randomized 657 patients to receive panitumumab plus platinum-based chemotherapy and 5-Fluorouracil, and patients could continue panitumumab until progression or unacceptable toxicity was observed. This treatment was tested *vs.* chemotherapy alone. The primary endpoint was overall survival (OS). The median OS was 11.1 months in the panitumumab arm and 9 months in the control arm (*p* = 0.14), and no statistically significant improvement was observed in the primary endpoint. Median progression-free survival (PFS) was 5.8 months in the panitumumab arm and 4.6 months in the control arm (HR 0.78 *p* = 0.0036), which was statistically significant but clinically modest. This trial also analysed HPV status by assessing p16 levels in the tumour samples of 443 patients. Of these, 22% were p16-positive and 78% were p16-negative. There was no difference in the median OS or PFS in patients who were p16-positive (OS: 11 months in the panitumumab arm and 12.6 months in the control arm; PFS: 5.6 months in the panitumumab arm *vs.* 5.5 months in the control arm). However, the p16-negative subgroup median OS (11.7 months in the panitumumab arm and 8.6 months in the control arm) and PFS (6 months *vs.* 5.1 months) were significantly longer, suggesting that p16 status could be a predictive biomarker for responsiveness in patients treated with panitumumab (or other anti-EGFR drugs). The most common adverse events registered in the trial that were associated with panitumumab were typical anti-EGFR side effects: skin toxicity, metabolic toxicity (including hypomagnesaemia or hypokalaemia), diarrhoea and cardiac arrhythmias.

These results are discordant in survival with those of the EXTREME trial [33] despite the fact that they share a similar design. Hypotheses that may explain this include: molecular differences between panitumumab and cetuximab, differences in the mechanisms of action (cetuximab can activate an antitumour immune response, but panitumumab does not), the type of population enrolled in both trials (most patients were from western Europe in the EXTREME trial, whereas in the SPECTRUM trial, most were from Eastern Europe and the Asia-Pacific region) or treatments prior to enrolment (38% received previous chemotherapy in the EXTREME trial, whereas 81% did so in the SPECTRUM trial). Although we do not know the cause of these differences, the fact is that panitumumab failed to improve OS in the phase III SPECTRUM trial.

*Zalutumumab* is a human monoclonal antibody against EGFR that has shown activity in preclinical models (both by blocking the EGFR signalling pathway and by stimulating an antitumour immune reaction by antibody-dependent cellular cytotoxicity (ADCC)).The results of a phase III trial [40] of this drug were published in 2011 to compare zalutumumab *vs.* best supportive care in patients with R/M HNSCC after the failure of platinum-based chemotherapy. The randomization was 2:1, with 191 patients in the zalutumumab arm and 95 in the control arm. The primary endpoint was OS, and the results were 6.7 months in the zalutumumab arm and 5.2 months in the control arm (*p* = 0.0648). The secondary endpoint was PFS, which was slightly better in the zalutumumab arm (9.9 *vs.* 8.4 weeks) (*p* = 0.0012). Rash was the most common toxicity (92%) in the experimental arm, followed by anaemia, pyrexia, diarrhoea and hypomagnesaemia. This study did not at all support the use of zalutumumab in R/M HNSCC after platinum progression, however new combination of zalutumumab could have been promising.

*Nimotuzumab* is a humanized anti-EGFR antibody that has been widely explored in local or locally advanced HNSCC alone or concurrent with radiotherapy, but there are no records indicating its use in R/M HNSCC. There is an ongoing phase II study of chemotherapy (cisplatin and 5-FU) combined With Nimotuzumab in untreated metastatic nasopharyngeal carcinoma (NCT01616849).

#### 3.1.6. Small Molecule Tyrosine Kinase Inhibitors (TKIs)

Erlotinib and gefitinib are oral small molecules that reversibly inhibit the tyrosine kinase (TK) activity of EGFR. Lapatinib is a reversible dual inhibitor of EGFR/Her-2 TK activity, and afatinib is an irreversible pan-inhibitor of the ErbB family. The first trials of these drugs explored the activity of the reversible TKIs (erlotinib and gefitinib) after first-line treatments for R/M HNSCC.

*Gefitinib* was compared to methotrexate at different doses (250 or 500 mg/day) in a phase III trial for R/M HNSCC [41]. The median OS was 5.6 months (gefitinib 250 mg), 6 months (gefitinib 500 mg) and 6.7 months (methotrexate), with no significant differences. The safety profiles were different: the most common adverse effects were stomatitis for methotrexate and skin reactions and diarrhoea for gefitinib. In conclusion, gefitinib did not improve survival in R/M HNSCC compared to methotrexate, and higher doses of gefitinib were not associated with better outcomes. Another strategy explored for gefitinib was its use in combination with chemotherapy (docetaxel) in a phase III trial by the Eastern Cooperative Oncology Group (ECOG) [42]. The dose chosen for gefitinib was 250 mg/day. However, the study did not meet the primary endpoint (OS): 7.3 months for the combination of gefitinib and docetaxel *vs.* 6 months for docetaxel alone (*p* = 0.6). The incidence of diarrhoea was higher for the combination arm.

For *Erlotinib,* there are no results from phase III trials in this setting because it showed limited activity in two previous phase II trials [43,44]. In one of these, erlotinib was used as a monotherapy at a dose of 150 mg/day in patients with R/M HNSCC after primary treatment. The overall response rate was 4.3%, and the median PFS was 9.6 weeks, although 38% of patients showed disease stabilization for 16 weeks. The very low response rate in erlotinib was improved by its combination with cisplatin (21%) in the other phase II trial, which showed a median PFS for these patients of 3.3 months. Currently, there is a randomized phase II study to test docetaxel and cisplatin with or without erlotinib in patients with R/M HNSCC.

*Lapatinib* has shown a lack of activity in R/M HNSCC. In a phase II trial [45] of a monotherapy dose of 1500 mg daily, there was no objective response in 45 of the patients. The best response was stable disease, which was observed in the cohort of patients that was never exposed to an anti-EGFR therapy, and the PFS was 52 days. Half of the patients experienced diarrhoea.

*Afatinib* (an irreversible pan-ErbB inhibitor), by contrast, showed better activity in a phase II trial [46] that compared it with cetuximab using a two-stage design (the first stage was a comparison of afatinib and cetuximab, and the second stage permitted crossover to the other therapy). The primary endpoint was mean tumour shrinkage during the first stage. In all, 121 patients began stage 1, and 68 began stage 2 (the principal reason for crossover was the progression of the disease). The doses were afatinib at 40 mg/day and cetuximab at 250 mg/m^2^/weekly. In the afatinib arm, the mean tumour shrinkage was assessed by an investigator (IR) and independent central review (ICR). The IR/ICR was 10.4%/16.6% for afatinib and 5.4%/10.1% for cetuximab (*p* = 0.46/0.30). The objective response rate was 16.1% (IR) for afatinib and 6.5% (IR) for cetuximab. The median PFS was comparable: 13 weeks (afatinib) and 15 weeks (cetuximab) by ICR. In the result of stage 2, the disease control rate by ICR was 33.3% for afatinib and 18.8% for cetuximab, and the PFS was 9.29 weeks in the afatinib arm and 5.71 weeks in the cetuximab arm (*p* = 0.077). Side effects were more common in the afatinib arm, especially for rash/acne, diarrhoea and stomatitis, but they were manageable. These results supported the use of a sequential anti-EGFR treatment for some HNSCC patients.

After this phase II trial, a trial for afatinib was developed in a programme called LUX-H & N [47]. Their recently published phase III trial (LUX-H & N1) included patients with R/M HNSCC after platinum-based therapy progression. A total of 483 patients were randomized to afatinib 40 mg/day (322 patients) or methotrexate 40 mg/m^2^ once weekly (161 patients). The primary endpoint was PFS, and the results were statistically positive: 2.6 months for afatinib *vs.* 1.7 months for methotrexate (HR 0.8 *p* = 0.03). However, we did not observe these differences in OS: 6.8 months for afatinib and 6 months for methotrexate. In the subgroup analyses, the benefit to PFS was greater in the afatinib arm for naïve EGFR inhibitor patients and for p16-negative tumours. Although this trial is positive in its primary endpoint, the lack of benefit to OS indicates that applying afatinib in routine clinical practice would be premature [48].

*Dacomitinib* (PF-00299804) is a novel irreversible pan-ErbB TK inhibitor. Its activity was explored in a phase II trial as a first line treatment in R/M HNSCC. A total of 69 patients were included, and they received dacomitinib at 45 mg/day. Only 63 patients were evaluable for ORR, which was 12.7% and therefore comparable to cetuximab as a monotherapy. Median PFS and OS were 12.1 weeks and 34.6 weeks, respectively. There were no surprises in its toxicity profile, which is similar to the results for the other anti-EGFR therapies previously mentioned [49,50].

### 3.2. VEGF Pathway: Targeting VEGF and PDGFR

VEGF is overexpressed is most HNSCCs, and it is related to poor outcomes in these patients. It has been shown that hypoxia stimulates proangiogenic factors and is associated with radiotherapy resistance. The therapeutic approaches against this pathway include monoclonal antibodies, such as bevacizumab (anti-VEGF), or multi-kinases inhibitors, such as sorafenib, sunitinib or vandetanib [51].

*Bevacizumab* is the monoclonal antibody with the most indications in medical oncology. This drug is approved for the treatment of metastatic breast cancer (not by the FDA), colorectal cancer, lung tumours, gynaecologic tumours (ovarian and cervix) and kidney cancer. The development of the drug in HNSCC has not been very successful due to the high risk of bleeding in these tumours. A phase II trial [52] was performed in combination with cetuximab in R/M HNSCC that excluded patients with tumours that invaded major vessels. The dose chosen for bevacizumab was 15 mg/kg. Among the 46 included patients, the ORR was 16% and the median PFS and OS were 2.8 months and 7.5 months, respectively. Even with the selection of patients, 12 of them suffered bleeding events of different grades. These results improved when the drug was combined with pemetrexed [53] (median PFS 5 months, median OS 11.3 months), but bleeding events were also worse (2 fatal events). Some questions that are pending with bevacizumab are: the optimal dose to minimize the risk of bleeding without jeopardizing efficacy and the search for a biomarker.

*Sunitinib* is an oral molecule that inhibits multiple TKIs (VEGFR-1, VEGFR-2, VEGFR-3, and PDGF) and that is approved for the treatment of renal carcinoma or GIST. However, its development in other tumours, such as breast cancer, has been stopped. The GORTEC group published a phase II trial of sunitinib at 37.5 mg daily in progressed HNSCC patients. The best response was stable disease (50%), and the median PFS and OS were very low (2–3.4 months). There was a high incidence (16%) of grade 3 to 5 bleeds [54].

*Sorafenib* is a multikinase inhibitor (VEGFR-2, VEGFR-3, PDGFR, and c-KIT) of tumour progression that acts through the MAPK family. The SWOG trial demonstrated a disease control rate of 51% (although PR was only 2%), and, interestingly, a median PFS of 4 months and a median OS of 9 months. Compared to sunitinib, the toxicity profile was more favourable for sorafenib (hand-food toxicity and diarrhoea) [55].

Here, we will briefly mention *Vandetanib* because a phase II trial that combined its use with docetaxel, drug with efficacy in head and neck cancer, did not show activity in R/M HNSCC patients [56]. There are currently ongoing trials for bevacizumab, including a phase III trial of chemotherapy with or without bevacizumab (NTC00588770), and sorafenib, including a phase I/II trial of sorafenib in combination with cisplatin and docetaxel (NTC02035527).

### 3.3. Targeting the PI3K Pathway

Many growth factor receptors are involved in the activation of the PI3K pathway [8,57], including EGFR, insulin-like growth factor receptor (IGFR) and FGFR. This activation triggers a cascade of signals that stimulates protein kinase B (AKT) and the mammalian Target of Rapamycin (mTOR). The PI3K/Akt/mTOR pathway is involved in the development of multiple tumours. Specifically, we have mentioned that mutations in PI3K are found in more than 30% of HNSCC (both HPV-negative and HPV-positive tumours); hence, while targeting this pathway has become a challenging, it serves a useful purpose.

To outline the results of these trials, the components of PI3K are a p110 catalytic subunit (p110α, p110β, p110δ) and a p85 regulatory subunit. To attack this pathway, different PI3K inhibitors have been developed, including mTOR inhibitors (temsirolimus or everolimus), Pan-PI3K inhibitors (buparlisib or PX-866) and p110α inhibitors (BYL719).

#### 3.3.1. mTOR Inhibitors

*Temsirolimus* (an analogue of rapamycin) has been explored in monotherapy and in combination with Erlotinib. The TEMHEAD trial recruited 40 patients with R/M HNSCC after platinum-based chemotherapy or cetuximab. Of those recruited, 57% of the patients achieved stable disease, and in 39% of the patients, the tumour shrunk. Both median PFS and OS were short (56 and 152 days, respectively). Toxicity is worth mentioning because almost 50% of the patients experienced fatigue, 25% experienced anaemia, and 20% experienced pneumonia [58].

The results of using temsirolimus in combination with erlotinib were even worse. Only 12 patients participated in this trial, which used a dose of 150 mg of erlotinib and 15 mg/weekly of temsirolimus. In total, 50% of the patients quit the treatment as a result of severe toxicity, which consisted on fatigue, diarrhoea, pneumonia and head and neck edema. The data for efficacy took a back seat because of these effects. The median PFS was 1.9 months [59].

*Everolimus* was tested in a phase II trial that followed the same design, with a dose of 5 mg/day in combination with erlotinib at 150 mg/day (R/M HNSCC) after platinum-based chemotherapy. Tolerance was better in this trial, and only one patient withdrew from treatment. ORR at 12 weeks was 2.8%, but 31% of the patients achieved stable disease. Median PFR was 11.9 weeks, and median OS was 10.25 months [60].

These three trials included a broad biomarker analysis (aimed at analysing EGFR, PI3KCA, PTEN, AKT, cKIT, and RAS, among others), but results were inconclusive because a predictive biomarker has not yet been identified.

#### 3.3.2. Pan-PI3K Inhibitors

*Buparlisib (BKM-120)*: The results of a Korean trial were presented at ASCO 2015. This trial included a clinical study (tolerance and efficacy of buparlisib) and a preclinical study (to enhance buparlisib activity in combination with other drugs). A total of 37 patients were enrolled, but the efficacy was modest: RR was 2.7%, median PFS was 7.4 weeks and OS was 19.2 weeks. Common toxicities included anorexia (62%) and hyperglycaemia (59%). The preclinical data showed inhibited cell growth when BKM120 was combined with cetuximab. This combination will be explored in an ongoing clinical trial (NTC01816984).

*PX-866* is another oral Pan-PI3K inhibitor that was explored in combination with cetuximab in a phase II randomized trial in patients after platinum-based progression (R/M HNSCC). A total of 83 patients were enrolled to receive weekly cetuximab or cetuximab in combination with PX-866 (8 mg/day). The combination did not improve ORR, PFS (80 days in both groups) or OS (211 days *vs.* 256 days), and toxicity was greater in the combination arm, especially gastrointestinal toxicity.

The same design was used in another phase II randomized trial that tested the drug combined with docetaxel (docetaxel plus/minus PX-866) in 85 patients, and the same results were observed. In summary: the efficacy results in terms of ORR, PFS or OS were not better for the combination, but toxicity was worse in that arm and included higher rates of febrile neutropenia [61,62].

In conclusion, although the role of the PI3K pathway is a promising target for drug development (proof of this hypothesis is the number of related ongoing clinical trials), the lack of biomarkers to select patients for these kind of treatments causes the results found in the clinical trials to be disappointing. We also cannot discount the high rates of associated toxicity. Perhaps with selected p110α-inhibitors, toxicity profiles will be improved.

### 3.4. Targeting Src

Src is a cytoplasmic tyrosine kinase that is associated with the proliferation or migration of tumour cells. Many growth factor receptors or G-protein-coupled receptors located in the plasma membrane can activate Src, including EGFR, VEGFR, PDGFD or FGFR. Consistent with the overexpression of EGFR in HNSCC, Src activity is also increased in these tumours. There is therefore a reason to develop inhibitors of Src tyrosine kinase to treat HNSCC.

*Saracatinib* (AZD0530) is an oral small molecule inhibitor of Src TK and Bcr-Abl. A total of nine patients were enrolled in a phase II trial after at least one prior therapy. The study was closed because eight of these patients progressed early (within the first 60 days). Toxicity was manageable, and fatigue was the most common adverse event [63].

*Dasatinib* (BMS-354825) is a multikinase inhibitor (Scr, PDGFR, BCR-ABL and c-Kit). A lack of activity for this drug was demonstrated in a phase II trial in a group of 15 patients. Only two of the patients showed disease stabilization, while seven patients progressed, and four patients withdrew because of toxicity (gastrointestinal effects and pleural effusion) [64]. Because of these results, blocking only Src in HNSCC is not the best strategy to explore in further clinical trials.

### 3.5. Promising Pathways

Alterations in NOTCH1 have been reported in more than 10% of HNSCCs. There are four transmembrane receptors (Notch1-4), and their ligands are Delta (δ1-3) and Jagged (Jag1-2). The ligand-receptor interaction stimulates proteolytic processes, including the following: ADAM protease splits the Notch extracellular domain, γ-secretase breaks the intracellular domain, and Notch is translocated to the nucleus, where it activates transcription factors such as HES, HERP, p21 or CDK-1. This pathway appears has been related to cell differentiation. There are currently no available drugs that work against this pathway [65,66].

The hedgehog (Hh) pathway is related to cell adhesion and epithelial-mesenchymal transition (EMT). Hh activates Gli1, which regulates many cancer genes (e.g., myc, VEGF or PTCH). Overexpression of the Gli1 protein can promote tumour growth and metastasis. A Hh inhibitor (IPI-926) is currently being explored in a clinical trial in R/M HNSCC (NTC01255800).

## 4. Inmmunotherapy

### 4.1. Introduction

The role of the immune system in the control of cancer has been suggested for decades by the observation of an increased incidence of malignancies in immune-suppressed [67] patients the prognostic role of the presence of T cells in some types of tumours [68,69] the presence of tumour-specific cytotoxic T cells in the peripheral blood, and the observation of rare spontaneous tumour regression.

In 1890, the first immunotherapy against cancer was developed by Coley based on the use of streptococcal toxins to raise immunity and improve the control of cancer [70].

Multiple immunologic strategies have been more recently tested. In recent years, advances in our knowledge of molecular biology, cancer immunology and drug development have made a number a therapeutic advances in cancer immunotherapy possible.

The immune system plays an important role in the development of head and neck cancer [71], which is shown especially in the fact that these tumours are consistently related to viruses, as in HPV-associated oropharyngeal and EBV-associated nasopharyngeal tumours. An impaired immune response appears to be the cause of cancer development in a minority of infected individuals [72]. A number of immunologic alterations have been observed in head and neck tumours, including the dysregulation of T cells [73], changes in cytokine production [74], deficiencies in the maturation of antigen-presenting dendritic cells [75] and the down-regulation of NK cells [76]. Although there seem to be some differences between HPV-positive and HPV-negative tumours, the activation of the PD1/PDL1 axis may be involved in T cell dysfunction in head and neck cancer regardless of its association with HPV infection. The increased expression of PDL-1 has been detected in head and neck cancer and in premalignant lesions [77,78].

The immune response has been shown to be prognostic in head and neck tumours as well in other tumour. Both T-cell infiltration [79,80,81] and the expression of immune checkpoint ligands and their receptors have been associated with prognosis, although this association has not been observed in other studies [82,83].

### 4.2. Immunotherapy Approaches

Immunotherapy refers to several treatments that have different mechanisms of action. All of them act to boost or restore immune function to fight cancer and other diseases.

Immunotherapy in head and neck cancer could have a role in all stages of the disease, from preventive vaccination against the viruses implicated in the development of head and neck tumours to the treatment of metastatic disease through the use of adjuvants. Currently, a wide variety of new immune-based cancer therapies are being developed for head and neck cancer patients.

Recently, promising results have been found in studies of PD-1 checkpoint inhibitors in metastatic patients. Future investigations will establish the benefit of these treatments in other stages of disease. Other immune checkpoints are currently being investigated. Their relevance in immune responses in cancer and the possible benefits of their inhibition or stimulation in the treatment of cancer are currently unknown. Other points that have not been answered include the question of why immunotherapy works in some patients but not in others who have the same cancer and the value of using immunotherapy in combination with other anticancer treatments, such as chemotherapy, radiation therapy or targeted therapies.

#### 4.2.1. Immune Checkpoint Modulation

The normal functions of the immune system include maintaining the right balance between activating and inhibiting proteins to avoid overly strong immune responses. These checkpoint proteins may be altered in cancer cells, which may help tumours to evade the immune system. Treatments targeting these proteins, either by blocking inhibitory signals or by stimulating positive signals, may restore an adequate immune response. Drugs that block specific immuno-checkpoint proteins have been successfully used to treat different cancer types. *Ipilimumab*, a monoclonal antibody that blocks the protein CTLA-4, was the first immune checkpoint modulator to be approved by the Food and Drug Administration (FDA) for the treatment of melanoma patients [84]. Ipilimumab is now being tested in many clinical trials in different cancer types, including squamous cell carcinoma of the head and neck (SCCHN) (NCT01860430, NCT01935921).

**Figure 4:**
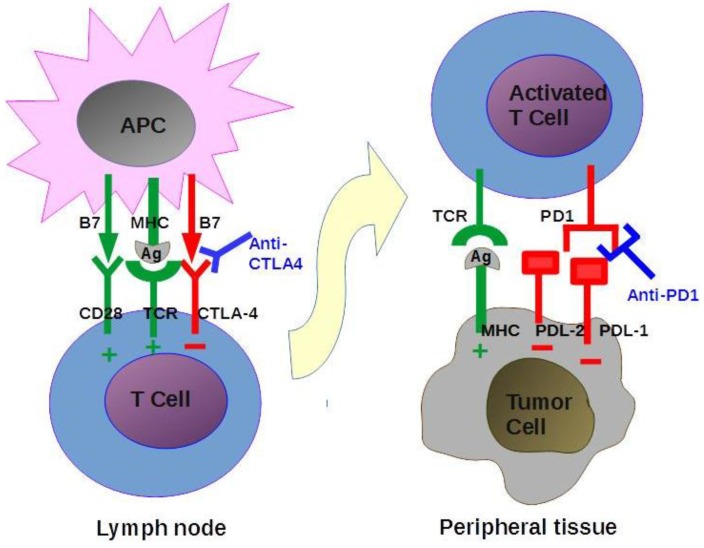
The interaction between T-cell receptors (TCRs) and major histocompatibility (MHC) molecules expressed on antigen-presenting cells (APCs) is the first event that leads to T cell activation. This occurs primarily in the lymph nodes. A subsequent interaction between the CD28 receptors on T-cells with B7 co-stimulatory molecules on APCs completes T cell activation. CTLA-4 and PD-1 are negative regulators of T cell immune responses, and they are required to avoid excessive immune activity. CTLA-4 binds to B7 and therefore competes with CD28. It seems to act early during T cell activation in the lymph nodes. PD-1 binds PDL-1 and PDL-2, which are both negative modulators of immune T cell responses. The role of PD-1 inhibitory activity seems to be more important in peripheral tissues during the effector phase. Anti-CTLA-4 and anti-PD-1 antibodies block these inhibitory checkpoints, thereby enhancing antitumour immune activity.

*Pembrolizumab* is a monoclonal antibody that targets the human programmed death receptor-1 (PD-1) by blocking its interaction with PDL-1 and PDL-2. This interaction inhibits the immune response; blockade of this pathway therefore causes an increased immune response, including antitumour immune responses. The inhibitory pathways involving CTLA-4 and PD1 are represented in Figure 4. Pembrolizumab was the first PD-1 inhibitor to be approved by the FDA for use in patients with advanced or unresectable melanoma [85]. Promising results for this drug were presented at the 2015 American Society for Clinical Oncology (ASCO) meeting from an expanded cohort of a phase I clinical trial testing the use of pembrolizumab in patients with recurrent or metastatic squamous cell carcinoma of the head and neck [86,87]. In this study, 132 pretreated patients received a fixed intravenous dose of 200 mg of pembrolizumab, regardless of their PD-L1 or HPV status. The overall response rate was 24.8%, with 57% of the patients showing some kind of decrease in the size of their target lesions and one patient achieving a complete response. Responses were observed in both human papillomavirus (HPV)-positive and HPV-negative tumours. Pembrolizumab was well tolerated. The most common observed side effects were fatigue, rash and pruritus. Serious immuno-related adverse events were observed in less than 10% of the patients.

Two ongoing phase III studies are evaluating pembrolizumab *vs.* standard treatment in patients with recurrent/metastatic head and neck cancer (NCT 02252042, NCT 02358031). Additional phase III studies of *nivolumab* (another anti-PD-1 antibody) and MEDI4736 (an anti-PD-L1 antibody) are underway for head and neck cancer. Combination studies of immune checkpoint inhibitors with cytotoxic chemotherapy or with molecularly targeted agents, such as inhibitors of the epidermal growth factor receptor or angiogenesis, are actively being conducted in different types of tumours.

The expression of PD-L1 has been proposed as a biomarker that may be a predictor of a benefit from anti-PD-1 agents. In a previous phase Ib trial, pembrolizumab produced an overall response rate of 20% in patients with advanced head and neck cancer, and a subgroup analysis showed a higher response rate (45.5%) in patients with tumours expressing PD-L1 above a cut-off point [88] (1% of staining in tumour cells or stroma) *vs.* 11.4% in those below the cut-off point. Similarly, preliminary data from a trial involving the administration of MEDI4736 to different tumour types showed an overall response rate of 14% in 22 patients with head and neck cancer, including a response rate of 50% in those whose tumours were PD-L1 positive *vs.* 6% in those whose tumours were PD-L1 negative [89]. The determination of multi-gene expression signatures has also been proposed as a predictive biomarker of response to pembrolizumab in head and neck cancer patients [87]. However, additional validation of these biomarkers is needed.

#### 4.2.2. Cytokine Therapies

Cytokines are intercellular regulatory proteins that play a crucial role in initiating, maintaining, and regulating immunologic homeostatic and inflammatory processes [90]. The therapeutic administration of cytokines or the modulation of their levels or activity or interference with their signal transduction pathways are promising candidates for therapeutic interference.

An example of this approach is *leukocyte interleukin*, a preparation of synthetic IL-1, IL-2, IL-6, tumour necrosis factor (TNF)-alpha, interferon gamma, and other stimulating molecules administered via peritumoural injection, which is currently been tested in a phase III trial for the treatment of oral cavity tumours (NCT01265849). The subcutaneous administration of *IRX-2*, a biological product that contains multiple cytokines produced after the stimulation of mononuclear cells obtained from normal healthy donors in a phase II trial.(NCT00210470) or the administration of recombinant IL-15 in a phase I clinical trial of solid tumours, including SCCHN (NCT01727076).

Toll-like receptors (TLRs) are proteins expressed in immune and non-immune cells [91], such as skin and oral mucosa cells. These have emerged as key mediators of immune functions. TLRs act as sensors by recognizing specific molecules. After their activation, TLRs initiate signalling pathways, leading to the release of cytokines and chemokines and the recruitment of different types of immune cells to the site of the tumour. Ten human TLRs (TLR-1 to TLR-10) have so far been identified, and they differ according to their cellular localization and difference in their activating ligands [92,93,94].

The addition of an agonist of TLR-9 to cetuximab failed to improve PFS in patients with recurrent or metastatic squamous cell cancer of the head and neck in a concluded phase II trial [95].

*VTX-2337* is a TLR-8 agonist that is currently under investigation in a phase II trial in patients with recurrent or metastatic SCCHN (NCT01836029). Previous data from a phase I clinical trial showed that the administration of VTX-2337 was clinically well tolerated and biologically active and that it showed a predictable pharmacokinetic profile [96].

#### 4.2.3. Adoptive T Cell Therapies

Adoptive T cell therapy is the transfusion of autologous or allogenic T cells into patients with the aim of enhancing antitumour immunity. There are many forms of adoptive T cell therapies being used for cancer treatment: culturing tumour-infiltrating lymphocytes (TILs), isolating and expanding one particular T cell or clone, and even using T cells that have been engineered to recognize specific tumour cells [97]. Studies have also tested T cells that have been removed from a patient and then stimulated or genetically modified to improve their activity before being re-introduced into the patient.

T cell therapy has been explored in many tumour types, where studies have achieved promising results in the treatment of melanoma patients [98]. This is also an attractive strategy for virus-induced cancers. Adoptive T-cell therapy directed against EBV antigens has met with some success for the treatment of EBV-associated nasopharyngeal cancers [99,100]. Preliminary studies have shown the feasibility of using an adoptive T-cell therapy that is directed against HPV-16 that might be successfully used to treat patients with HPV-associated cancers [101]. A variety of studies have been and are now being conducted to investigate a potential role of this kind of immunotherapy in EBV-related nasopharyngeal cancer. The feasibility of Epstein-Barr virus-specific adoptive T-cell immunotherapy is also being tested (NCT00431210). A phase I clinical trial of TGF-beta-resistant cytotoxic T-lymphocytes for the treatment of EBV-positive nasopharyngeal carcinoma is ongoing (NCT02065362). In patients with HPV-associated cancer, the infusion of tumour-infiltrating lymphocytes is being investigated in a phase II trial (NCT01585428).

Examples of adoptive T-cell therapy in initial clinical trials in head and neck tumours have been documented. Autologous T4-positive T cells engineered to express a specific antigen receptor are being administered to head and neck patients in a phase I clinical trial (NCT01818323). NCT02239861 is a clinical trial for patients with solid tumours, including head and neck cancer, in which specific cytotoxic T lymphocytes against tumour specific antigens are being used.

#### 4.2.4. Cancer Vaccines

Prophylactic vaccination against high-risk HPV types has been shown to prevent a significant number of cervical carcinomas [90,91,92,93,94,95,96,97,98,99,100,101,102]. These vaccines might also reduce the incidence of HPV-associated oropharyngeal cancers as well as other HPV-related cancers. The presence of HPV-16 DNA in a relatively high fraction of HPV-associated HNSCC suggests [103] that the currently available vaccines may be particularly effective in preventing this disease, although this effect will need to be determined in the future.

Therapeutic HPV vaccination strategies are also being developed. This approach, known as antigen-specific immunotherapy, relies on the induction of a cellular immune response that is able to both prevent and produce the regression of neoplastic lesions. Viral proteins E6 and E7 are consistently expressed in most HPV-associated cancers, and they therefore represent good targets for the development of vaccines aimed at controlling HPV-mediated cancers.

Several clinical trials, including trials including HPV-HNSCC patients, are currently underway that are based on encouraging pre-clinical results involving preventive and therapeutic HPV vaccines [104]. A phase II clinical trial of ADXS11-001, a vaccine designed to induce an immune response against the viral oncoprotein E7, is underway in HPV-positive oropharyngeal cancer patients (NCT02002182). Pre-clinical studies have demonstrated the successful reduction of *in vivo* tumour burden in animal cancer models [105]. There are also clinical trials combining conventional therapy and vaccinations HPV-positive cancer patients, including the NCT02526316 clinical trial that combines cisplatin-based chemotherapy with a P16 peptide vaccine. Previously reported data [106] have shown that the administration of this vaccine is safe and well tolerated in patients with HPV-related cancer.

## 5. Conclusions

Platin-based chemotherapy it’s the standard treatment in LA-RMSCCHN, today only cetuximab has been approved in this disease other than locally advanced and recurrent head and neck cancer, however there are different drugs under development. The major problem with SCCHN is the heterogeneity of these group of tumors, with a lot of primary tumors, different molecular pathway and probably different approach. Until now EGFR has been the principal target with respect to development of new drugs, but might be necessary to know other alternative to the control of recurrent/metastatic disease and locally advanced stage. Probably the development of immunotherapy in few times with randomized trial could offer another alternative, but in our opinion, all new drugs under development needs to be compare with platin-base regimen and probably with other regimen that include cetuximab such as Extreme and Cetuximab-Rt, however with respect to combination with radiotherapy, cisplatin-Rt it’s the gold standard treatment. In medicine , only randomized phase III trial could change the standard regimen and in our review we demonstrated a lot of phase II trial but there are a few phase III at the moment. Probably the design of study with similar population characteristics such as HPV+ or HPV−, primary tumor localization, inclusive recurrent or metastatic disease, can offer changes in these complex group of diseases.
